# Main controls on *phoD*-harboring bacterial community abundance, composition, and activity from oil-contaminated soils at the Changqing oilfield, Northwest China

**DOI:** 10.3389/fmicb.2025.1732638

**Published:** 2026-01-13

**Authors:** Dandan Li, Xianyuan Du, Xingchun Li, Xinyu Zhang, Jin Zheng, Qin Chu, Weiwen Qiu, Hanyu Zhang, Qingwei Wang

**Affiliations:** 1 Institute of Carbon Neutrality, Key Laboratory of Sustainable Forest Ecosystem Management-Ministry of Education, School of Ecology, Northeast Forestry University, Harbin, China; 2 Key Laboratory of Ecosystem Network Observation and Modeling, Institute of Geographic Sciences and Natural Resources Research, Chinese Academy of Sciences, Beijing, China; 3 State key Laboratory of Petroleum Pollution Control, CNPC Research Institute of Safety and Environmental Technology, Beijing, China; 4 Key Laboratory of Saline-alkali Vegetation Ecology Restoration, Ministry of Education, College of Life Science, Northeast Forestry University, Harbin, China; 5 College of Resources and Environment, University of Chinese Academy of Sciences, Beijing, China; 6 The New Zealand Institute for Plant and Food Research Limited, Christchurch, New Zealand

**Keywords:** total petroleum hydrocarbons, oil contamination, alkaline phosphatase activity, phoD-harboring bacteria, microbial community, Changqing oil field

## Abstract

**Background:**

Soil phosphorus (p) availability limits the native microbial activity, which then inhibits the petroleum hydrocarbon biodegradation. Microbial communities harbouring the alkaline phosphatase (ALP) *phoD* gene (*phoD*-harboring bacteria community, hereafter) play the key roles in the regulation of P availability in soils. Nevertheless, the consequences of oil contamination on ALP activity and *phoD*-harboring bacterial community dynamics are poorly understood. It is necessary to assess *phoD*-harboring bacterial abundance, community diversity, and ALP activity in response to oil contamination. This information would be useful for formulating plans for future bioremediation processes.

**Methods:**

In this study, we sampled the contaminated and uncontaminated soils in the area surrounding crude oil pumping wells at the Changqing oilfield. The Real-time Quantitative PCR (qPCR) was used to detect the abundance of *phoD* gene. The diversities and compositions of *phoD*-haboring microbial communities were illustrated via Illumina high-throughput sequencing. Coincident soil chemical properties (soil water content (SWC), total petroleum hydrocarbons (TPHs), total nitrogen (TN), soil organic carbon (SOC), total phosphorus (TP), nitrate (NO_3_^-^-N), ammonium (NH_4_^+^-N), dissolved organic carbon (DOC), available phosphorus (AP)) and ALP activities were also quantified.

**Results:**

We observed that petroleum contamination markedly decreased the abundance, richness, and diversity of the *phoD*-harboring bacterial community but greatly enhanced the relative abundance of *phoD*-harboring Actinomycetia, Thermoleophilia, and Rubrobacteria (*p* < 0.05). The relative abundances of *phoD*-harboring Alphaproteobacteria, Betaproteobacteria, and Gammaproteobacteria showed an increasing tendency and then decreased as the oil contamination concentration increased (*p* < 0.05). The soil water, nutrient content [carbon (C), nitrogen (N), and phosphorus], and nutrient ratio were the crucial parameters influencing the *phoD*-harboring bacterial community responding to oil contamination. The activity of ALP was associated positively and negatively with the relative abundance of Betaproteobacteria and Rubrobacteria, respectively.

**Discussion:**

Overall, the oil pollution stress altered the abundance, richness and composition of the active *phoD*-harboring functional microbial community. A significant decline in ALP activity in the oil-contaminated soils was likely caused by reduced abundance and changes in the composition of the *phoD*-harboring bacterial community, which were strongly dependent on the available N and P contents.

## 1 Introduction

As a major energy source in contemporary society, the role of oil in the growth of national economies is crucial. With the wide utilization of oil products, however, soil contamination caused by oil has become a severe concern for the environment (Chakravarty et al., 2022). Microbial degradation is considered to be a major pathway in the removal of oil from soils (Koshlaf et al., 2020; Ou et al., 2025). Bioremediation using indigenous microbes is the preferred approach to remediate petroleum-contaminated soils (Zhao et al., 2011), owing to its cost-effectiveness, simplicity of operation, and environmental friendliness (Liu H. et al., 2020). Microbial growth may be phosphorus-limited, as it depends on phosphorus for synthesizing nucleic acids and proteins (Esberg et al., 2009). Even though petroleum hydrocarbons in the soils provided considerable sources of carbon (C) for microorganisms, the relative deficiency of phosphorus nutrition can restrict microbial activity and suppress petroleum hydrocarbon biodegradation (Li et al., 2022a; Yu et al., 2024).

Soil microorganisms are capable of mineralizing organic phosphorus to inorganic phosphates through the secretion of phosphatases (Dai et al., 2020; He et al., 2025) that are categorized as acid and alkaline phosphatases depending on their optimal pH (Luo et al., 2019). Acid phosphatase is generated by plants, animals, and microorganisms, while alkaline phosphatase (ALP) is primarily derived in soil from bacteria (Wang et al., 2021). Compared to acid phosphatase, ALP has become widely used to assess the mineralization of organic P in neutral and alkaline soils (Hu et al., 2018; Liu S. et al., 2020). ALP is encoded by the *phoD* gene, which exists ubiquitously in high abundances in various soil types and bacterial taxa (Ragot et al., 2015; He et al., 2025; Xu et al., 2025). The P accessibility highly controlled the expression of the *phoD* gene (Ragot et al., 2016; Lang et al., 2020; Wang et al., 2022). In general, it is possible that the *phoD*-harboring bacteria may start contributing to maintaining the availability of P when mineral P becomes exhausted in the soil (Vershinina and Znamenskaya, 2002; Wei et al., 2021). Consequently, understanding the abundance, composition, and activity of the *phoD*-harboring bacterial community responding to petroleum contamination is crucial for mediating phosphorus availability and facilitating the bioremediation of petroleum-contaminated soils.

Several studies have indicated that soil physicochemical property parameters play significant roles in regulating the spatial distribution of *phoD*-harboring bacterial communities in uncontaminated locations (Chen et al., 2023; Li W. et al., 2023; Xu et al., 2025). The decreased soil pH was closely related to the decreased abundance of *Alphaproteobacteria*, *Gammaproteobacteria*, and *Actinobacteria* containing *phoD* genes (Dai et al., 2020). Electrical conductivity strictly limited the *phoD* gene expression in the soils contaminated with heavy metals (Lu et al., 2022). Luo et al. (2019) concluded that soil N: P and C: P ratios became the major factors in predicting the *phoD*-harboring bacterial community abundance and diversity. Compared to P-adequate soils, a significant increase in ALP activity was observed from relatively P-deficient soils (Xu et al., 2022), which was confirmed by the finding that ALP was generally generated by the bacteria during the period of phosphate starvation (Gardner et al., 2014). Frankia was a critical group for the detection of P deficiencies, generating ALP and increasing P availability in the soils of arid and semiarid regions (Xu et al., 2022). Chen et al. (2023) emphasized that soil organic carbon stability shaped the *phoD*-harboring bacterial community and enhanced biological processes of soil phosphorus. Petroleum pollutants caused changes to soil physicochemical properties to varying degrees. Petroleum contamination in soil can decrease water and oxygen content and permeability (Devatha et al., 2019). Hydrocarbon compounds in petroleum also significantly boost the contents of soil carbon and change the soil nutrient ratio (Yuan et al., 2022; Song et al., 2025). To date, it is still unknown how the variations in soil physicochemical property parameters induced by petroleum contamination affect the *phoD*-harboring bacterial community dynamics.

Numerous researchers documented significant changes of microbial community structures in the soils contaminated with oil (Khan et al., 2018; Wang et al., 2019; Li et al., 2022b). Petroleum hydrocarbon in the soils altered the functional distribution of soil microbial communities. In the contaminated soils from the Daqing and Huabei oil fields, the hydrocarbon-degrading groups increased, while the nitrogen-transforming groups decreased (Liao et al., 2015). It had also been reported that microorganisms involved in phosphorus cycling, including *Mycobacterium*, *Pseudomonas*, *Xanthomonas*, *Micrococcus*, *Sclerotium*, *Bacillus*, *Fusarium*, *Aspergillus*, and *Penicillium*, exhibited a higher sensitivity in the oil-contaminated soils (Chikere et al., 2011). Under crude oil contamination conditions at a concentration of 0.5%, the activity of ALP decreased after 30 days of cultivation (Dindar et al., 2015). This inhibitory phenomenon was likely to be caused by the suppression of microbial communities involved in the phosphorus cycle (Dindar et al., 2015). However, it is unclear how oil pollution influences alkaline phosphatase activity by regulating the *phoD*-harboring bacterial community.

Changqing oilfield, situated on the Loess Plateau in the provinces of Shaanxi, Gansu, and Ningxia, is an ecologically vulnerable area because of the factors of desertification and soil erosion (Yuan et al., 2022). The Changqing oilfield is one of the major oil-producing regions and creates over 500 hm^2^ of oil contamination (Li et al., 2022a). The depletion of soil bioavailable N and P was attributed to the growth of heterotrophic microorganisms that utilize a large quantity of carbon sources originating from total petroleum hydrocarbon (Gao et al., 2022). Therefore, we hypothesized that (1) the population and activity of the *phoD*-harboring bacterial community will increase because of increased P limitation to microbial metabolism, leading to improvement of P availability in the soils contaminated with low-concentration crude oil; (2) total petroleum hydrocarbons (TPH), total soil nitrogen, and total soil phosphorus contents would be key factors in shaping the abundance, diversity, and composition of the *phoD*-harboring bacterial community and the activity of ALP. To test these hypotheses, we collected contaminated soil and pristine uncontaminated soil within a 1-square-kilometer area surrounding crude oil pumping wells in the Changqing oilfield to assess the effects of petroleum pollutants on soil *phoD*-harboring bacterial abundance and community structure and ALP activities. We also determined the primary factors controlling *phoD*-harboring bacterial community and ALP activities via analyzing the physicochemical characteristics of oil-contaminated field soils. This study was conducted to offer new insights into microbial phosphorus cycling in the contaminated soil of oil fields, with the aim of contributing to the biological remediation of oil spills.

## 2 Materials and methods

### 2.1 Site description and soil collection

The research location was in the Changqing oilfield (34° 20′N, 107° 10′E) in Qingyang County, Gansu Province, Northwest China. It has a temperate continental monsoon climate with an approximate average annual precipitation of 470 mm and an average annual temperature of 10.7 °C (Liang et al., 2011). Soil types are yellow loamy soils (Yuan et al., 2022).

The sampling spots are located at the 2nd Oil Production Plant of the Changqing oilfield. The area where we collected soil samples is bare land without vegetation cover. In May 2022, soil samples were collected using 2-cm diameter augers within the area of 1 square kilometer surrounding the pumping well in the Changqing oilfield (Supplementary Figure S1). Contaminated (S2, S3, and S4) and uncontaminated (S1) soils were sampled from three directions along pumping wells. The site at S1 was not affected by production activities and served as the control. The separation between each sampling site in the same direction was over 100 m. In each sampling site (10 m × 10 m), 10 soil core samples (0–10 cm) were randomly selected to form one soil sample. Three replicate samples were obtained from sampling sites in all three directions at equal distances from the pumping well site and transported to the lab in an icebox. After being passed over a sieve of 2 mm to discard visible organic debris, soil samples were grouped into three subsamples for the extraction of DNA, the analysis of ALP activity, and the determination of soil physicochemical properties, respectively.

### 2.2 Soil physicochemical property and ALP activity

A test of soil physicochemical properties was carried out as described by Bao (2008). Soil water content (SWC) and pH were measured by the gravimetric and electrode methods, respectively. Extraction of dissolved organic carbon (DOC), available nitrogen (AN: NO_3_^−^ and NH_4_^+^), and available phosphorus (AP) from soils was performed using deionized water, 2 M KCl, and NaHCO_3_ (pH 8.5), respectively. Extraction of total phosphorus (TP) from soils was performed using an acid solution (H_2_SO_4_-HClO_4_). Soil total nitrogen (TN) was measured by the dry combustion method. Soil organic carbon (SOC) was estimated by oxidation with dichromate. Extraction of total petroleum hydrocarbons (TPHs) in the soil was performed using an acetone and dichloromethane mixture (1:1, V: V; Li et al., 2020). The contents of TPHs were assayed by the gravimetric method after solvent evaporation using a nitrogen blowing instrument (Yuan et al., 2022). The activities of ALP (E. C.3.1.3.1) were measured using a fluorometer (Synergy^H4^, BioTek, United States) as described by Saiya-Cork et al. (2002). The Supplementary material provided detailed methods for the determination of ALP activity.

### 2.3 *phoD*-harboring bacterial abundance, diversity, and community structure

Microbial DNA was obtained as described by Li et al. (2018). Assessment of *phoD* gene abundance was performed by quantitative PCR using a thermocycler analyzer (Eco™, Illumina, United States) with primers for F733/R1083 (Chen et al., 2019). Assessment of the *phoD***-**harboring bacterial diversities and community structures was performed with a sequencing platform of Illumina MiSeq PE300. Sequence data were placed in the database of the NCBI Sequence Read Archive (SRA) under the accession number PRJNA908779. The Supplementary material provided detailed methods for quantitative PCR and Illumina MiSeq sequencing.

Merged sequences were obtained by processing the raw sequence using FLASH (Version 1.2.11) and then filtering it with QIIME (Version 1.9.1) to discard ones of low quality. The clustering of sequences into operational taxonomic units (OTUs) was executed at a 97% similarity (Edgar, 2013). The classification of each sequence was performed on the basis of information from the Functional Gene Database (FunGene), applying a 0.75 confidence threshold (Maidak et al., 2001; Chen et al., 2019). The coverage indices (Supplementary Table S2) and the rarefaction curves (Supplementary Figure S2) indicated that the data volumes of the sequencing reads were reasonable. Mothur (version 1.30.2) was used to calculate the *α*-diversity indices of the *phoD*-harboring bacterial community (Simpson, Shannon, Abundance-based Coverage Estimator, and Chao). The relative abundance of the *phoD*-harboring bacterial community was the ratio of the sum of OTUs belonging to a homogeneous phylogenetic group to the whole OTU number.

### 2.4 Statistical analysis

Values were means ±standard deviations (*n* = 3). In order to satisfy the criteria of normal distribution, the abundances of *the phoD* gene have been log-transformed. One-way ANOVA and Duncan’s test were used to analyze the differences in physicochemical property parameters (TPH, SWC, pH, SOC, TN, TP, DOC, AN, and AP), gene abundance, diversity indices, *phoD* community relative abundance, and ALP activity among the sampling sites. The relationships between gene abundance, diversity indices, ALP activity, and physicochemical parameters were investigated by SPSS (version 27.0, IBM SPSS, Inc.) using Pearson’s correlation with a 95% confidence interval.

The following analysis was performed using R (Version 3.3.1). Changes in *phoD* community structures were evaluated using a Bray–Curtis distance matrix through principal coordinate analysis (PCoA). Significant differences in the community composition of *phoD* among oil-contaminated soils were assessed on the basis of permutational multivariate analysis (PERMANOVA) with the “adonis” function (Anderson, 2001). Correlations between the community structure of *phoD* and physicochemical property factors were identified using canonical correspondence analysis (CCA) and the Mantel test by the “vegan” package.

The indirect and direct effects of physicochemical properties and *phoD* community characteristics on ALP activity were examined using structural equation modeling (SEM), based on the method of maximum likelihood in AMOS 26.0 (IBM, Chicago, United States). Non-significant χ^2^ tests (*p* > 0.05), root mean square error of approximation (RMSEA, *p*< 0.05), and goodness-of-fit index (GFI, > 0.9) were considered acceptable. The bootstrapping procedure was applied to extend sample size (1,000 bootstrap samples) due to the small sample size (Li D. et al., 2023). The first axis of the PCoA score was applied to proxy *phoD* community structures (Li et al., 2021).

## 3 Results

### 3.1 The *phoD* gene abundance

**Figure 1:**
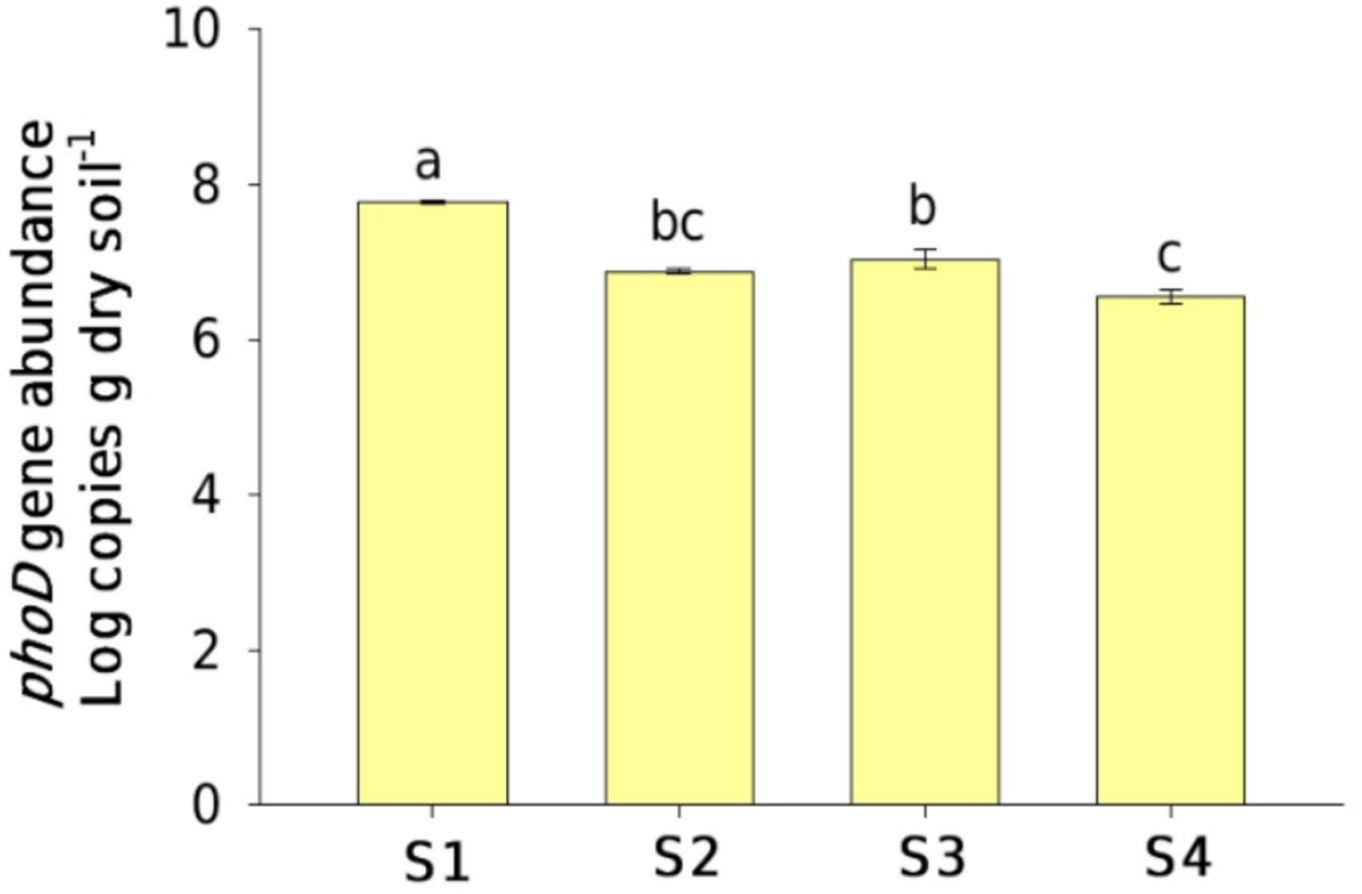
Changes in the *phoD* gene abundance in oil-contaminated soils at the Changqing oilfield. Lowercase letters indicate significant differences (*p* < 0.05) among the sampling sites.

Across the four sampling sites, *phoD* gene abundance varying from 3.7 × 10^6^ ~ 4.7 × 10^7^ g^−1^ dry soil was significantly different (Figure 1). The *phoD* gene abundance at site S1 was approximately 13 times higher than those at site S4 (*p* < 0.05). Positive correlations were found between SWC, NH_4_^+^, and AP contents and *phoD* gene abundance, and negative correlations were found between pH, NO_3_^−^, and TPH contents and *phoD* gene abundance (*p* < 0.05; Table 1).

**Table 1:** Correlations between the *phoD* gene abundance, the Chao index, the Shannon index, and soil properties (*n* = 12).

Factor	*phoD* gene abundance	Chao index	Shannon index
TPH	−0.82**	−0.59*	−0.67*
pH	−0.82**	0.36	0.46
SWC	0.90**	0.62*	0.66*
SOC	−0.34	−0.38	−0.22
NH_4_^+^	−0.73**	−0.27	−0.33
NO_3_^−^	0.76**	0.24	0.28
AN	0.64*	0.06	0.02
AP	0.74**	0.30	0.31
AN/AP	0.11	−0.73**	−0.81**
DOC/AP	0.11	−0.75**	−0.81**

TPH, total petroleum hydrocarbon; SWC, soil water content; SOC, soil organic carbon; NH_4_^+^, ammonium nitrogen; NO_3_^−^, nitrogen nitrate; AN (NH_4_^+^ + NO_3_^−^), available nitrogen; AP, available phosphorus; DOC, dissolved organic carbon. *p < 0.05; **p < 0.01.

### 3.2 Alpha diversity of the *phoD*-harboring bacterial community

**Figure 2:**
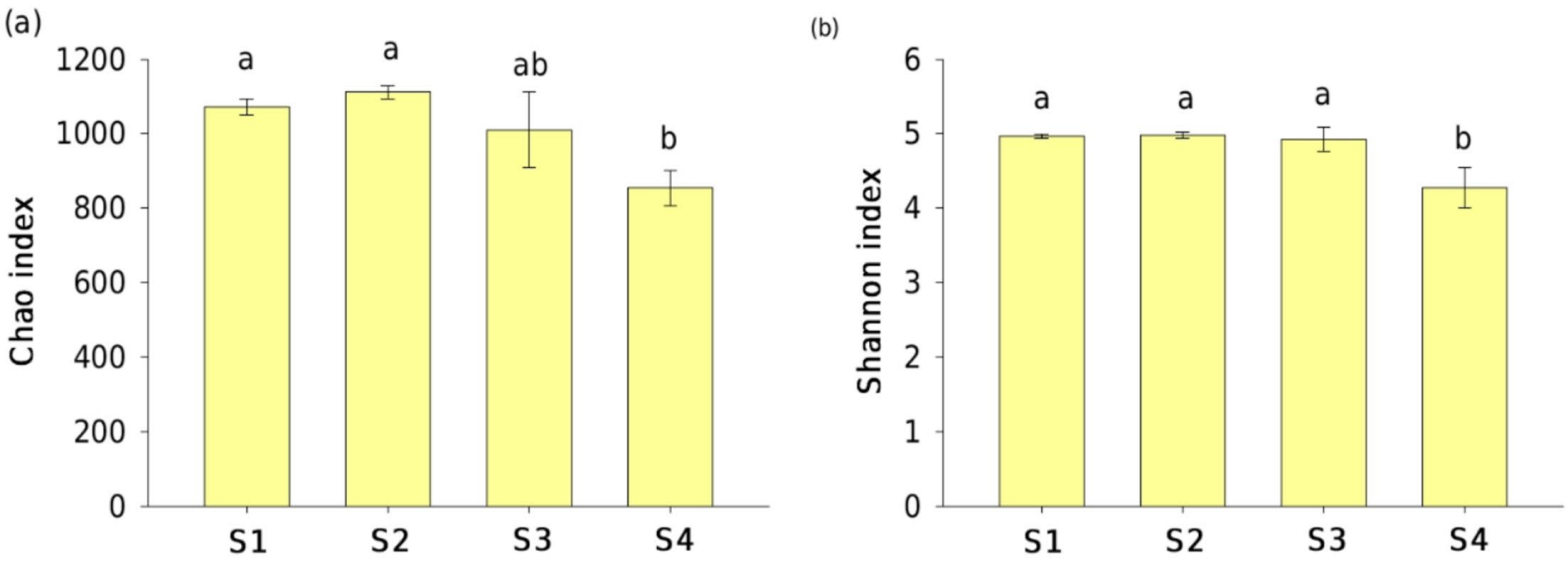
OTU-level **(a)** community richness (Chao index) and **(b)** diversity (Shannon index) of *phoD*-harboring bacteria in oil-contaminated soils at the Changqing oilfield. Lowercase letters indicate significant differences (*p* < 0.05) among the sampling sites.

The Chao and Shannon indices were used to estimate the *phoD*-harboring bacterial community richness and diversity, respectively. High values of the Chao and Shannon indices indicate the high community richness and diversity, respectively. Both the Chao and Shannon indices were similar among S1, S2, and S3, all of which had significantly larger values than those in S4 (*p* < 0.05; Figures 2a,b). The Chao and Shannon indices were positively related to SWC and NH_4_^+^ contents but were negatively related to nutrient stoichiometry (AN/AP and DOC/AP), TPH, and NO_3_^−^ contents (Table 1).

### 3.3 *phoD*-harboring bacterial community composition

**Figure 3:**
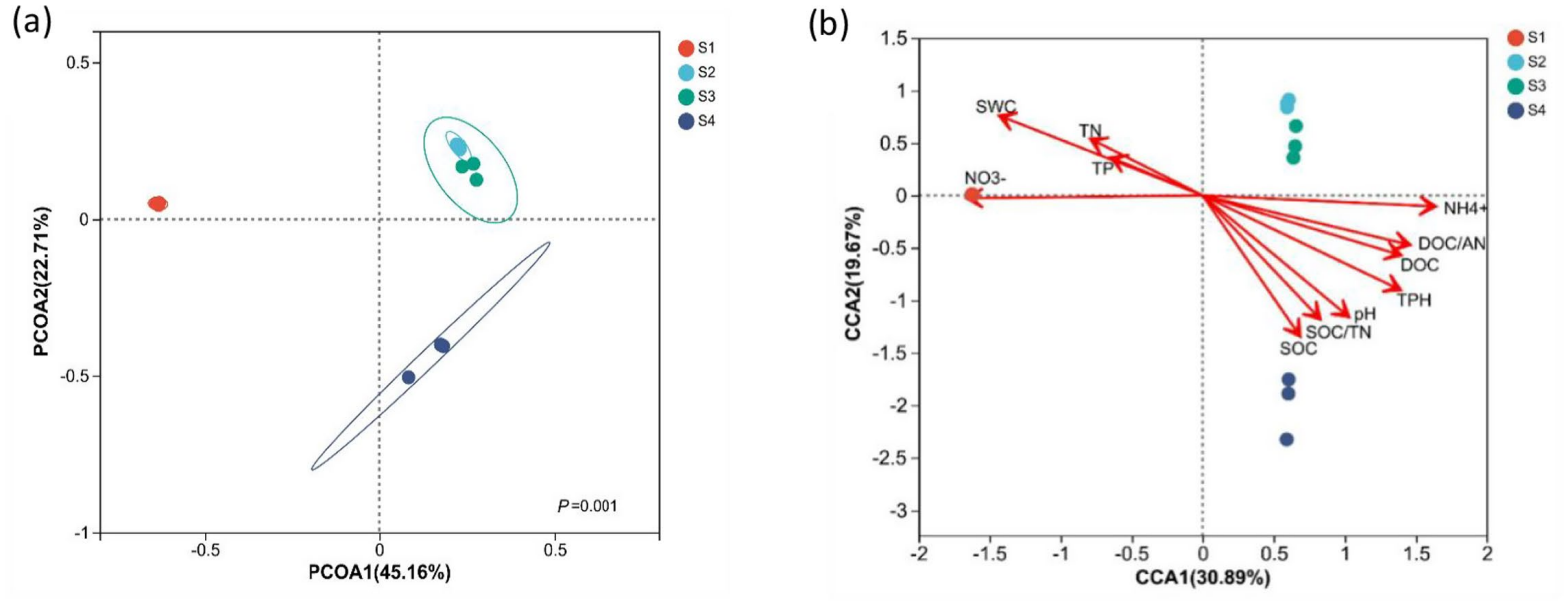
**(a)** Principal coordinate analysis (PCoA) of the *phoD*-harboring bacterial community among the oil-contaminated soils at the Changqing oilfield. **(b)** Canonical correspondence analysis (CCA) of the *phoD*-harboring bacterial community compositions in the oil-contaminated soils at the Changqing oilfield. The abbreviations are the same as those used in Table 1 (*n* = 12).

The compositions of *the phoD*-harboring bacterial **c**ommunity were similar in S2 and S3 and were separated from the sites of S1 and S4 (Figure 3a). PERMANOVA analysis showed different *phoD*-harboring bacterial community composition in oil-contaminated soil at Changqing oilfields (Figure 3a). The CCA showed that soil physicochemical properties together accounted for 51% of the variance in the composition of this *phoD*-harboring bacterial **c**ommunity composition (Figure 3b). The key soil physicochemical property parameters affecting shifts in the composition of the *phoD*-harboring bacteria community were the SWC, TN, TP, SOC, AN, and TPH contents and nutrient stoichiometry (SOC/TN and DOC/AN; Figure 3b; Supplementary Table S2).

**Figure 4:**
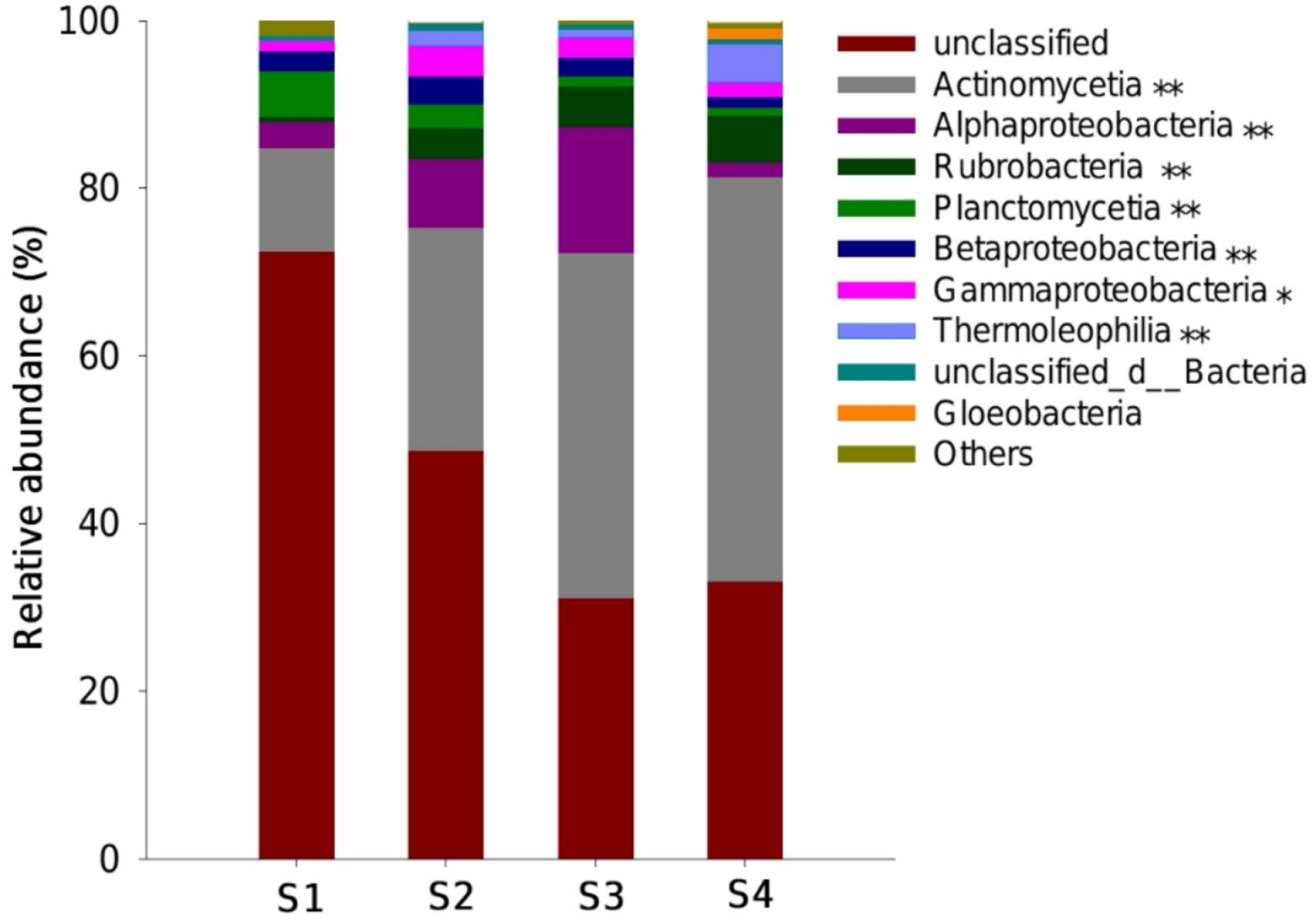
Relative abundance of *phoD*-harboring bacterial communities at the class level among the oil-contaminated soils at the Changqing oilfield. The groups accounting for < 1% are combined as others. The * and ** within the legend represent significant differences at *p* < 0.05 and *p* < 0.01 levels, respectively.

The predominant *phoD*-harboring bacterial communities detected in all samples included *Actinomycetia*, *Alphaproteobacteria*, *Rubrobacteria*, *Planctomycetia*, *Betaproteobacteria*, *Gammaproteobacteria*, *Thermoleophilia,* and *Gloeobacteria* (Figure 4). The most abundant functional groups were *Actinomycetia* among the *phoD*-harboring bacterial communities and increased from 13% in S1 to 48% in S4 (*p* < 0.01; Figure 4). Both *Thermoleophilia* and *Rubrobacteria* abundance increased from the sites of S1 to S4. By contrast, the *Planctomycetia* showed a decreasing tendency from the sites of S1 to S4. Compared to S1, *Alphaproteobacteria*, *Betaproteobacteria,* and *Gammaproteobacteria* increased in the sites of S2 and S3 and then decreased in S4 (Figure 4). *Actinomycetia, Thermoleophilia,* and *Rubrobacteria* abundances were positively related to TPH, SOC, and DOC contents but were negatively related to NO_3_^−^, AN, TN, AP, and TP contents (*p* < 0.05; Supplementary Figure S3). Both *Alphaproteobacteria* and *Betaproteobacteria* abundances were positively related to NO_3_^−^, AN, TN, AP, TP, and DOC but were negatively related to TPH contents (Supplementary Figure S3).

### 3.4 Alkaline phosphatase activity

**Figure 5:**
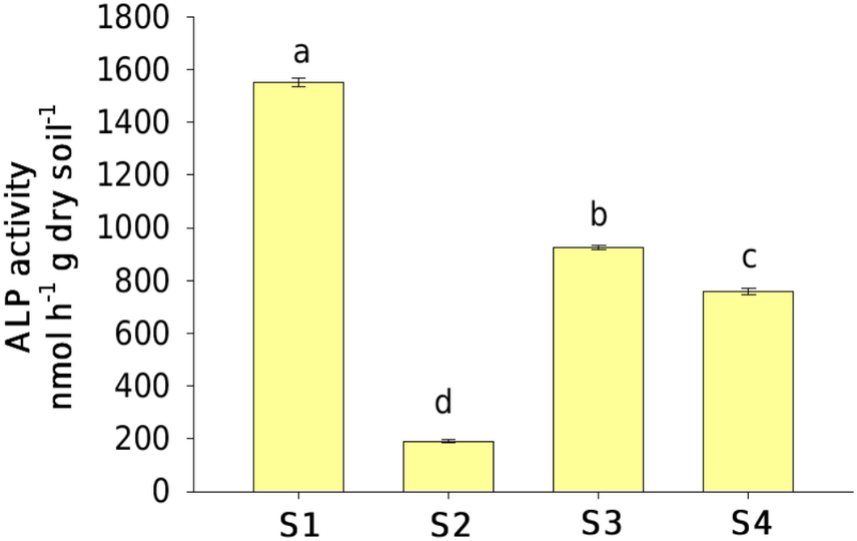
Alkaline phosphatase (ALP) activities in the oil-contaminated soils at the Changqing oilfield. Lowercase letters indicate significant differences (*p* < 0.05) among the sampling sites.

**Figure 6:**
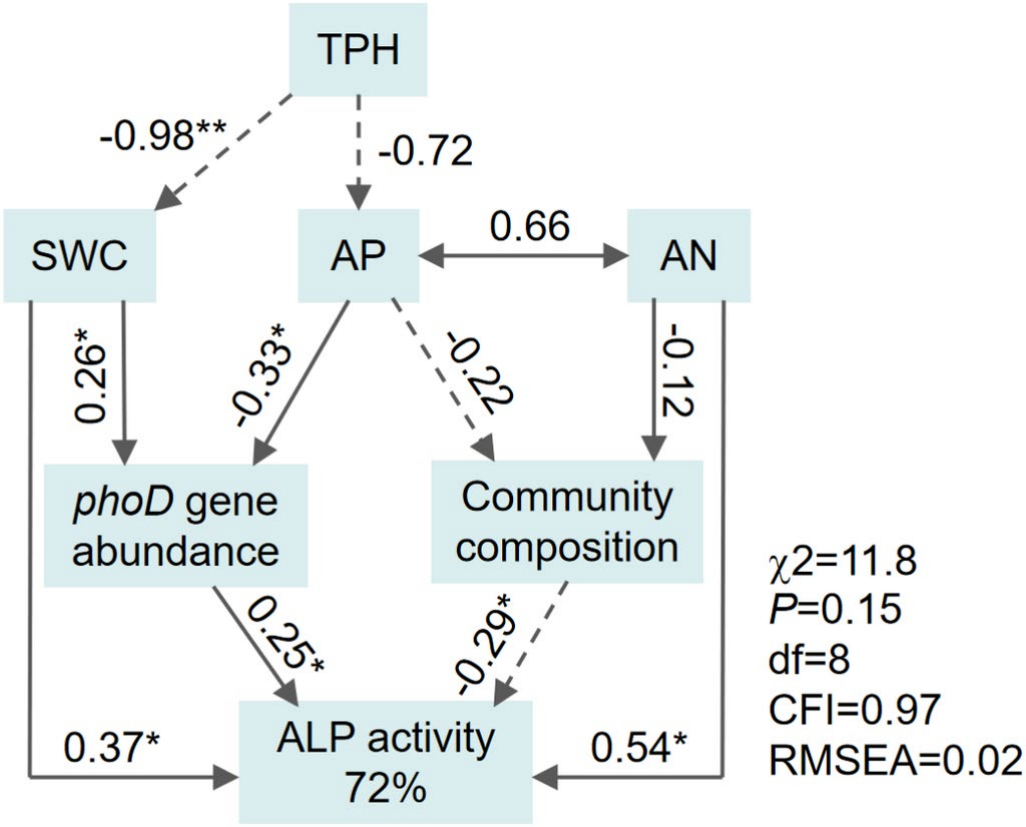
Structural equation model (SEM) describing the direct and indirect influences of soil physicochemical properties and *phoD*-harboring bacterial community characteristics on alkaline phosphatase (ALP) activity. The dashed and solid lines represent negative and positive path coefficients, respectively (*p* < 0.05). The *phoD* community composition was described by PCoA1.

The activities of ALP in the contaminated sites (90 ~ 924 nmol h^−1^ g dry soil^−1^) were significantly lower than those in the uncontaminated site (1,550 nmol h^−1^ g dry soil^−1^) (Figure 5). ALP activity was positively associated with SWC, AN, and AP contents; *phoD* gene abundance; and *Betaproteobacteria* abundance but was negatively associated with TPH content, *phoD* community structure, and *Rubrobacteria* abundance (*p* < 0.05; Table 2). The SEM explained 62% of variations in the activities of ALP (Figure 6). The comprehensive factors, including SWC, AP, and AN contents, *phoD* gene abundance, and community structures, exerted effects on the activities of ALP. The activities of ALP were directly affected by AN (path coefficient = 0.54), SWC (path coefficient = 0.27), *phoD* gene abundance (path coefficient = 0.25), and *phoD*-harboring bacterial community structures (path coefficient = −0.29) and were indirectly affected by TPH and AP content.

**Table 2:** Correlations between the alkaline phosphatase activities, soil physicochemical properties, and *phoD*-harboring bacterial community characteristics.

Variables	Factors	*R*	*p*
Physicochemical property	TPH	−0.560	0.052
	SWC	0.695*	0.012
	AN	0.802**	0.002
	AP	0.803**	0.002
Microbial characteristic	*phoD* gene abundance	0.794**	0.002
	*phoD* gene community composition	−0.821**	0.001
	*Rubrobacteria*	−0.584*	0.046
	*Betaproteobacteria*	0.836**	0.001

TPH, total petroleum hydrocarbon; SWC, soil water content; SOC, soil organic carbon; NH_4_^+^, ammonium nitrogen; NO_3_^−^, nitrogen nitrate; AN (NH_4_^+^ + NO_3_^−^), available nitrogen; AP, available phosphorus; DOC, dissolved organic carbon. *p < 0.05; **p < 0.01.

## 4 Discussion

### 4.1 Effects of oil contamination on *phoD*-harboring bacterial community abundance and diversity

We compared the *phoD* gene abundance and *phoD*-harboring bacterial community diversities in oil-contaminated soils with three levels of TPH content to undisturbed pristine soils. The TPH content of the soils was ranked as follows: S1 < S2 ≈ S3 < S4 (*p* < 0.05; Supplementary Table S1). The results demonstrated that *phoD* gene abundance and *phoD*-harboring bacterial community diversities were negatively impacted by the TPH content, as obvious declines in *phoD* gene copies and Chao and Shannon indices in the contaminated soils were observed. The correlation analysis also illustrated negative relationships between functional gene abundance, *α*-diversity indices, and TPH content (Table 1). These findings were inconsistent with our first hypothesis, which suggested an increased population of the *phoD*-harboring bacterial community in the soils contaminated with oil. A decrease in abundance and diversity could be attributed to the toxic oil components (Nejidat et al., 2023; Yu et al., 2024). The findings were similar to previous studies by Li et al. (2022a), who suggested that petroleum pollution can significantly reduce bacterial and fungal abundance in the Changqing oilfield. However, oil pollution greatly enhanced bacterial abundance, richness, and biodiversity in the Huabei and Daqing oil fields (Liao et al., 2015). Megharaj et al. (2000) showed that a stable microbial abundance was observed in the low petroleum pollution group (< 2,100 mg TPH kg^−1^ soil). This study found a considerable decrease in microbial abundance at the sampling sites of S2, S3, and S4, where the TPH concentration ranged from 554 to 999 mg kg^−1^. In contrast to previous studies, a relatively low concentration of petroleum would trigger a substantial reduction in the number of microorganisms involved in the phosphorus cycle. Furthermore, both the Chao and Shannon indices did not show differences at the sites of S1, S2, and S3, suggesting that a higher threshold concentration of TPH was essential to result in changes in diversity compared to absolute abundance at this field site.

Spatial heterogeneity of soil physical and chemical characteristics leads to notable differences in soil microbial community abundance and diversity responding to oil contamination in regions with different geographic locations (Liu et al., 2009; Sutton et al., 2013; Song et al., 2025). It was found that SWC showed a positive correlation with *phoD* gene abundance and *phoD*-harboring bacterial community diversities (including the Shannon and Chao indices) at the study sites (Table 1). Water is widely recognized as a major influencing factor for the activities of soil microorganisms, having a crucial contribution to the distribution of *phoD*-harboring bacterial communities in uncontaminated ecosystems (Li W. et al., 2023; Xu et al., 2025). Generally, an increase in TPH content in soils could enhance soil hydrophobicity that reduces SWC and water storage capacity (Supplementary Table S4), and this causes a further limitation of the connectivity between nutrient-rich soil micropores and immobilizing soluble substrates in the soil matrix (Khamehchiyan et al., 2007; Devatha et al., 2019; Marañón-Jiménez et al., 2022). In this study, we present the observation that a decreased *phoD* gene abundance in the petroleum-contaminated soils was associated with decreased soil water and nutrient contents (AN and AP) despite abundant carbon sources. Moreover, the Changqing oilfield is situated in arid and semiarid regions, which receive very low annual rainfall. The water availability in this area, prone to drought or scarce water resources, has a major effect in dictating changes in the *phoD*-harboring bacterial community in oil-contaminated soils. The *phoD* gene abundance was positively dependent on NO_3_^−^ content and negatively dependent on the NH_4_^+^ content, possibly because P and N transformation processes were found to be highly related, as reported by several previous researchers (Liu S. et al., 2020; Xu et al., 2022). Furthermore, the Chao and Shannon indices varied significantly depending on the ratios of AN/AP and DOC/AP in the soils post-oil contamination (Table 1), indicating that these bacteria carrying the *phoD* gene directly regulated species richness and community diversity rather than their proliferation in reaction to the soil nutrient stoichiometry imbalance, which was caused by a great quantity of carbon input originating from hydrocarbon.

### 4.2 Effects of oil contamination on *phoD*-harboring bacterial community composition

There were considerable variations in *phoD*-harboring bacterial community composition from oil-contaminated soil compared to undisturbed pristine soil (Figure 3), suggesting that the oil contamination concentration is a major factor reshaping the distribution of *phoD* microorganisms in the Changqing oilfield. Therefore, the high similarity of *phoD*-harboring bacterial community composition at the sites of S2 and S3 may be explained by the non-significant TPH content in these two sites. Apart from THP content, other soil physicochemical properties, i.e., SWC, TN, TP, SOC, and AN contents and nutrient ratio, were also important factors influencing distribution of the community structures. The above results indicated that the inputs of hydrocarbon significantly altered the soil physicochemical properties (Supplementary Table S4) and consequently altered *phoD*-harboring bacterial community compositions (Zhou et al., 2017).

Although the abundance and diversity of the overall *phoD*-harboring bacterial community have declined, the community composition has undergone a dramatic change, with the enrichment of tolerant groups and the decline of sensitive groups within it. In this study, it was observed that the relative abundance of *Actinobacteria*, *Rubrobacteria,* and *Thermoleophilia* increased from the sites of S1 to S4. Both *Rubrobacteria* and *Thermoleophilia* detected in this study belong to a separate subline of *Actinobacteria*. Previous studies reported that *Actinobacteria* were prevalent in oil-contaminated environments and were the dominant group of *phoD*-harboring microbes within different soil environments (Huang et al., 2021; Lu et al., 2022). These findings suggested that *Actinobacteria* may be strongly adapted to the environment contaminated by petroleum in the study sites. The resource competition theory highlighted that species with low trophicity are the only ones that are selected by resource limits (Tilman, 1982). The most abundant *Actinobacteria* were observed in oil-contaminated soils, which may be due to the fact that these bacteria have a trophic lifestyle similar to oligotrophic microorganisms (Dai et al., 2020; Wei et al., 2021). We indeed observed that *Acidobacteria* showed a negative correlation with the contents of N and P in the soils, which further supports our second hypothesis that N and P nutrients are key factors influencing *phoD*-harboring bacterial community composition. In general, oil contamination affects microbial survival and growth in soil, causing changes in microbial community composition by forcing native soil microbes to adapt to the new environment and elevating the enrichment of hydrocarbon-tolerant microorganisms (Calvo et al., 2019).

By contrast, *Alphaproteobacteria*, *Betaproteobacteria,* and *Gammaproteobacteria* were categorized in the *Proteobacteria* phylum, which was primarily derived from copiotrophic microorganisms (Dai et al., 2020). Compared to oligotrophs, copiotrophic bacteria had a fast growth in response to available C concentration (Luo et al., 2019) and highly demanding N and P nutrients (Dai et al., 2020). In this study, the *Proteobacteria* phylum showed positive correlations with DOC, AN, and AP contents. The high level of available C input derived from hydrocarbons led to the enrichment of the copiotrophs (*Proteobact*eria) under low contamination stress, while the growth of these groups is also dependent on other nutrient availability (e.g., N and P) under high contamination stress. In the present study, the observed relative abundance of *Alphaproteobacteria*, *Betaproteobacteria,* and *Gammaproteobacteria* tended to increase and then decrease from the sites of S1 to S4. This trend confirmed the previous observation that microbial population increased as TPH concentration increased, possibly due to the adaptation of soil microorganisms to lower-dose TPH (Guo et al., 2012).

### 4.3 Understanding the drivers of alkaline phosphatase activity in oil-contaminated soils

This study found that ALP activities in contaminated soil were lower than those in uncontaminated soil, which was opposite to our first hypothesis. Previous studies by other authors reported that either a significant decline or increase in microbial activity appeared in the soils after oil pollution (Meng et al., 2018; Chakravarty et al., 2022; Song et al., 2025). Low levels of P availability would evoke the expression of phosphatases to meet requirements for bacterial growth in infertile soils, resulting in an increase in ALP activities (Liu et al., 2021; Luo et al., 2021). The suppression observed in our study, however, was most likely caused by the inhibition of beneficial microbial populations due to oil contamination (Telesiński et al., 2018; Chakravarty et al., 2022). A toxic effect for petroleum hydrocarbon decreases the total abundance of the *phoD*-harboring bacterial community, as it reduces the availability of substrate for microorganisms (as described above), thereby ultimately impeding the enzymatic reactions (Chakravarty et al., 2022). In this study, a significant decline in ALP activity was observed as TPH concentration increased, which was directly affected by the decreased abundance and altered *phoD*-harboring bacterial community compositions. Surprisingly, *phoD*-harboring community compositions had negative impacts on ALP activity, which could be attributed to different lifestyle characteristics that the species in *phoD* functional groups usually possess (e.g., copiotrophs and oligotrophs). The overall ALP production capacity of this new community was lower than that of the original community following soil oil contamination, although the unit efficiency of some of its groups might be very high.

Among the different *phoD*-harboring bacterial community members, the activity of ALP showed positive and negative correlations with the relative abundance of copiotrophic *Betaproteobacteria* and oligotrophic *Rubrobacteria*, respectively (*p* < 0.05). Xu et al. (2025) have reported that not all microorganisms harboring the *phoD* gene play a crucial role in the synthesis of ALP. Xu et al. (2025) and the current findings suggest that total alkaline phosphatase production was the result of the interaction among different taxa that harbor similar amino acid sequences but possess contrasting lifestyle characteristics. Furthermore, both *Betaproteobacteria* and *Rubrobacteria* were in low abundance, only accounting for under 4% of all *phoD* gene sequences, and were considered to be rare taxa. Both *Betaproteobacteria* and *Rubrobacteria*, two rare microbial taxa, played crucial roles in maintaining soil ecosystem functions, according to a growing number of studies (Xu et al., 2022). Our previous studies also observed that rare microbial taxa were more closely associated with the activities of ALP than the abundant ones (Liu S. et al., 2020). The present findings further highlighted the importance of rare *phoD*-harboring microorganisms in the promotion of ALP activity from oil-contaminated soils. *Betaproteobacteria* have been reported to be found and isolated from the majority of contaminated soils, and many of them were confirmed as petroleum degradation agent pioneers. This finding infers that some *Betaproteobacteria*, as petroleum hydrocarbon degraders, are capable of mineralizing organic phosphorus to release bioavailable phosphorus. Therefore, increasing the abundance of *Betaproteobacteria* that engage in both hydrocarbon degradation and organic-P mineralization in oil-contaminated soils could improve soil nutrient conditions and benefit the survival and proliferation of these microbes. Our study demonstrates that the presence of culturable *Betaproteobacteria* will facilitate future *in situ* bioremediation of oil-contaminated soils in the Changqing oil field.

## 5 Conclusion

This is the first study to investigate *phoD*-harboring bacteria community abundance and composition in response to oil contamination. Oil contamination concentration was found to have negative influences on *phoD* gene abundance and *phoD*-harboring bacteria community richness and diversity due to the decline in water and nutrient contents in the oil-contaminated soils. The composition of *the phoD*-harboring bacteria community altered because of oil contamination, with *Actinomycetia* being the predominantly abundant indicator species. The increasing abundances of *Actinomycetia, Thermoleophilia,* and *Rubrobacteria* were also observed in the oil-contaminated soil. *Alphaproteobacteria*, *Betaproteobacteria,* and *Gammaproteobacteria* abundance tended to increase and then decrease as oil contamination concentration increased. In addition, ALP activity in contaminated soil appeared to be reduced compared to uncontaminated soil. The copiotrophic *Betaproteobacteria* and oligotrophic *Rubrobacteria* played great roles in maintaining ALP activity of the oil-contaminated soils. Furthermore, the rare *Betaproteobacteria* was engaged in both organic-P mineralization and hydrocarbon degradation in the soils with oil contamination and would be fully utilized to enhance petroleum hydrocarbon biodegradation.

## Data Availability

The datasets presented in this study can be found in online repositories. The names of the repository/repositories and accession number(s) can be found in the article/Supplementary material.
