# Supplementary material for: Main controls on phoD-harboring bacterial community abundance, composition, and activity from oil-contaminated soils at the Changqing oilfield, Northwest China
Source: Front Microbiol. 2026 Jan 13;16:1732638. doi: 10.3389/fmicb.2025.1732638 (PMC12835899; doi:10.3389/fmicb.2025.1732638)
Supplement: Supplementary file 1 [file Table_1.DOCX]

**Supplementary Material**

**Alkaline phosphatase activity assay**

The ALP (E.C.3.1.3.1) activity was measured with reference to Saiya-Cork et al. (2002). The average pH value of soil samples at this study sites was 8.3, hence we set to use a buffer with a pH of 8.8 to determine ALP activity. The 4-methylumbelliferone-phosphate was selected as the substrate. Briefly, soil suspending solution was prepared by adding 1 g fresh soil to 125 mL buffer solution and was homogeneously mixed for 1 min. The 200 μL soil suspending solution and 50 μL substrate (200 μM) were added to the 96-well plates, and then was incubated at 25 °C for 4 h. The activity of ALP was estimated by fluorometer (Synergy^H4^, BioTek, USA). A total of eight replicate wells per sample was conducted in this study.

**Quantitative PCR of *phoD* gene**

Soil microbial genomic DNA was extracted using DNeasy PowerSoil Pro Kit (Qiagen, Hilden, Germany). The concentration and purity of extracted DNA was then determined using a NanoDrop ND-2000 spectrophotometer (Nanodrop Technologies, USA). The abundance of *phoD* gene were evaluated by a thermocycler (Eco™, Illumina, USA) using the primer pairs F733/R1083 (Chen et al., 2019). The reaction system contained 1 μL of DNA template, 0.8 μL of each primer (5 μM), 7.4 μL of sterile water and 10 μL of TaKaRa Premix (TaKaRa, Japan) (Li et al., 2018). Bovine serum albumin was also added to suppress potential amplification inhibitors in the soil. The PCR procedures were as follows: 95 °C for 2 min, 35 cycles of 95 °C for 30 s, 50 °C for 30 s and 72 °C for 1 min. The standard curves were established using a dilution series of up to 10-fold of PCR products containing fragments with known target gene copy numbers. The amplification efficiencies ranged from 95 to 105%, and R2 value was greater than 0.996.

**Illumina MiSeq sequencing**

The community structure and diversity of *phoD*-harboring bacteria were evaluated using an Illumina MiSeq PE300 (paired end 300) sequencing platform (Illumina, San Diego, USA). The PCR was performed with specific primers F733 (5′′-barcode-TGGGAYGATCAYGARGT-3′) / R1083 (5′-CTGSGCSAKSACRTTCCA-3′) for 3 replicates of each sample. The PCR products of the same sample were mixed, and then puriﬁed using AxyPrepDNA Gel Extraction Kit (Axygen, USA). QuantiFluor™-ST (Promega) was used for the quantification of DNA. Finally, the purified PCR products were pooled in equimolar concentrations for Illumina Miseq sequencing (Illumina, Inc., San Diego, CA, USA) following the standard protocol of the processing laboratory (Majorbio Bio-Pharm Technology Co. Ltd., Shanghai, China). The sequence data were deposited into the NCBI Sequence Read Archive (SRA) database (Accession Number: PRJNA908779).

**References**

Saiya-Cork, K.R., Sinsabaugh, R.L., Zak, D.R., 2002. The effects of long term nitrogen deposition on extracellular enzyme activity in an Acer saccharum forest soil, Soil Biology and Biochemistry 34, 1309-1315.

Chen, X., Jiang, N., Condron, L.M., Dunfield, K.E., Chen, Z., Wang, J., Chen, L., 2019. Soil alkaline phosphatase activity and bacterial phoD gene abundance and diversity under long-term nitrogen and manure inputs. Geoderma 349, 36-44.

Li, D., Zhang, X., Green, S.M., Dungait, J.A.J., Wen, X., Tang, Y., Guo, Z., Yang, Y., Sun, X., Quine, T.A., 2018. Nitrogen functional gene activity in soil profiles under progressive vegetative recovery after abandonment of agriculture at the Puding Karst Critical Zone Observatory, SW China. Soil Biology and Biochemistry 125, 93-102.


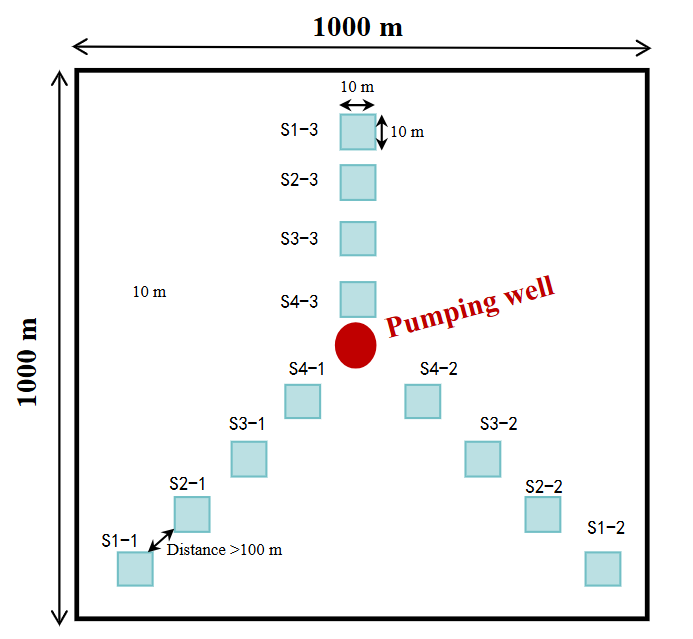


**Fig. S1** Schematic diagram of sampling sites distribution in the study.


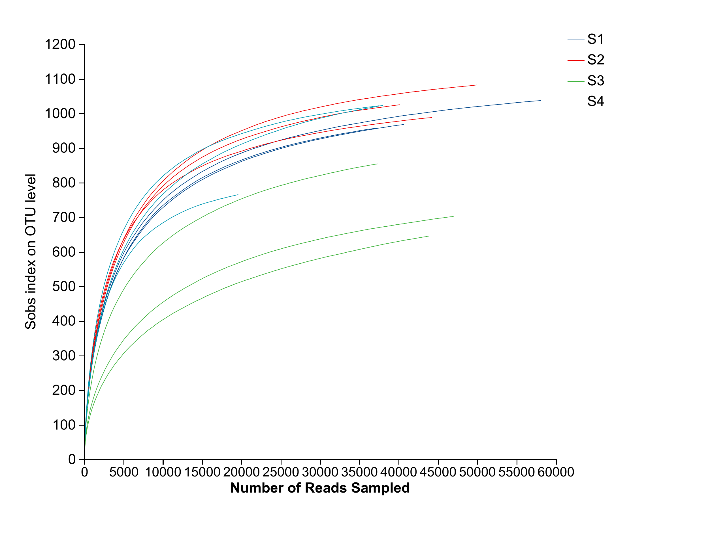


**Fig. S2** The rarefaction curve analysis of the *phoD*-harboring bacterial sequences.


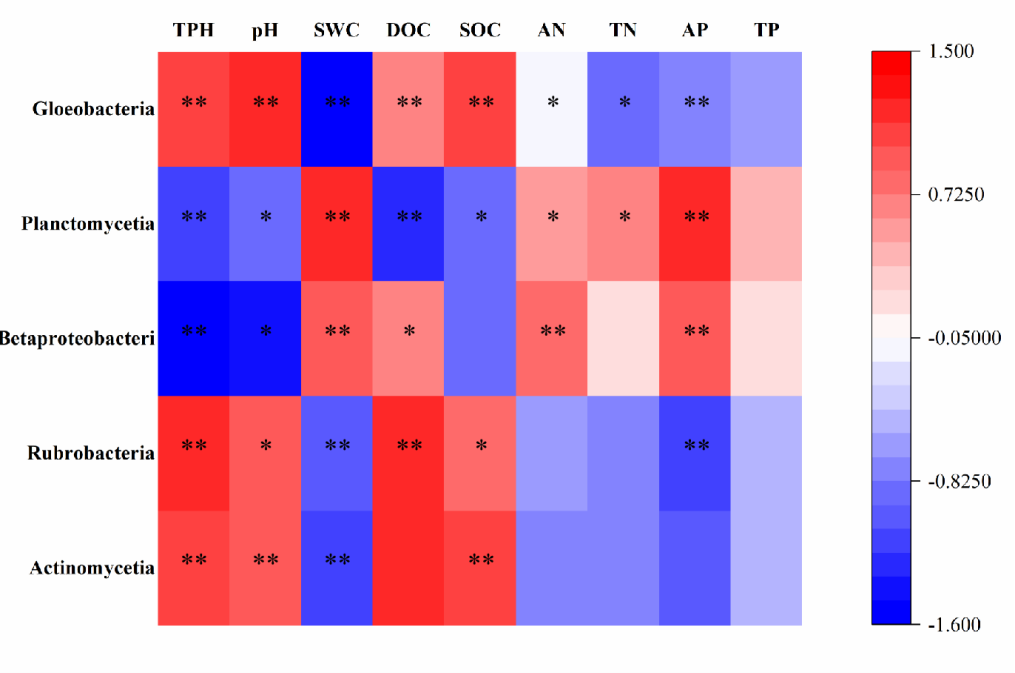


**Fig. S3** Pearson’s correlation coefficients between the soil physicochemical properties and the relative abundance of dominant *phoD*-harboring bacterial groups. **P* < 0.05; ** *P* < 0.01. The abbreviations are the same as those used in Table 1.

**Table S1** Soil physical and chemical properties in Changqing oilfield. Different letters represent signiﬁcant differences among samples at the level of *P* < 0.05. The abbreviations are the same as those used in Table 1

|  | S1 | S2 | S3 | S4 |
| --- | --- | --- | --- | --- |
| TPH (mg kg^-1^) | 11.8±1.1c | 554.2±21.7b | 597.6±18.1b | 999.7±48.1a |
| pH | 7.62±0.03d | 8.35±0.04b | 7.93±0.04c | 9.32±0.09a |
| SWC (%) | 13.47±0.05a | 6.07±0.04c | 7.80±0.03b | 1.71±0.01d |
| NO_3_^-^ (mg N kg^-1^) | 25.80±0.75a | 4.12±0.15b | 4.52±0.22b | 4.87±0.16b |
| NH_4_^+^ (mg N kg^-1^) | 2.47±0.20b | 19.13±1.48a | 20.60±1.04a | 20.94±0.71a |
| AN (mg N kg^-1^) | 28.27±0.91a | 23.25±1.35b | 25.11±0.53b | 25.80±0.15ab |
| TN (g kg^-1^) | 1.20±0.36a | 1.13±0.42a | 0.40±0.06a | 0.42±0.07a |
| TP (g kg^-1^) | 0.30±0.020a | 0.28±0.01a | 0.04±0.001a | 0.08±0.02a |
| AP (mg N kg^-1^) | 22.18±3.56a | 2.60±0.40b | 0.33±0.02b | 3.02±0.24b |
| DOC (mg N kg^-1^) | 93.33±1.22c | 255.46±7.14b | 472.17±8.00a | 482.34±7.95a |
| SOC (g kg^-1^) | 10.67± 0.20c | 7.32±0.41d | 22.14±1.04b | 34.62±0.89a |
| SOC/TN | 10.81±0.56c | 9.36±0.71c | 57.09±3.75b | 88.46±10.73a |
| TN/TP | 10.00±1.80a | 4.83±0.15a | 10.47±0.91a | 4.99±0.55a |
| SOC/TP | 99.82±10.45b | 52.95±5.93b | 591.94±31.52a | 441.81±31.02a |
| DOC/AN | 3.31±0.13c | 11.03±0.65b | 18.81±0.75a | 18.70±0.72a |
| AN/AP | 0.55±0.02b | 9.38±0.52b | 77.67±9.38a | 8.65±0.18b |
| DOC/AP | 1.80±0.11b | 103.32±12.10b | 1465.42±133.30a | 161.58±11.93b |

**Table S2** Alpha-diversity of *phoD*-harboring bacteria in the oil-contaminated soils

| Sites | Sobs | Simpson index | ACE index | Good’s coverage |
| --- | --- | --- | --- | --- |
| S1 | 987 | 0.028 | 1051 | 0.999 |
| S2 | 1032 | 0.028 | 1085 | 0.999 |
| S3 | 734 | 0.033 | 999 | 0.998 |
| S4 | 937 | 0.038 | 844 | 0.998 |

**Table S3** Mantel test between the *phoD*-harboring bacterial community composition and soil properties in the oil-contaminated soils at Changqing oilfield. *P* < 0.05 indicated significant effects. The abbreviations are the same as those used in Table 1

| **Factor** | *R* | *P* |
| --- | --- | --- |
| **TPH** | 0.54** | 0.002 |
| **pH** | 0.77** | 0.001 |
| **SWC** | 0.78** | 0.001 |
| **AN** | 0.72** | 0.001 |
| **TN** | 0.75** | 0.001 |
| **TP** | 0.78** | 0.001 |
| **AP** | 0.43** | 0.004 |
| **DOC** | 0.25* | 0.021 |
| **SOC** | 0.67** | 0.001 |
| **DOC/AN** | 0.30* | 0.017 |

**Table S4** Correlation analysis for soil properties in this study (n=12)

| Parameter | TPH | PH | SWC | AN | TN | TP | AP | DOC | SOC |
| --- | --- | --- | --- | --- | --- | --- | --- | --- | --- |
| TPH | 1 | 0.748** | -0.804** | -0.497 | -0.775** | -0.329 | -0.599 | 0.839** | 0.706* |
| PH | 0.748** | 1 | -0.916** | -0.483 | -0.393 | 0.049 | -0.217 | 0.608* | 0.371 |
| SWC | -0.804** | -.916** | 1 | 0.490 | 0.414 | -0.084 | 0.196 | -0.622* | -0.399 |
| AN | -0.497 | -0.483 | 0.490 | 1 | 0.464 | 0.329 | 0.531 | -0.406 | 0.203 |
| TN | -0.775** | -0.393 | 0.414 | 0.464 | 1 | 0.729** | 0.340 | -0.520 | -0.535 |
| TP | -0.329 | 0.049 | -0.084 | 0.329 | 0.729** | 1 | 0.427 | -0.350 | -0.322 |
| AP | -0.399 | -0.217 | 0.196 | 0.531 | 0.340 | 0.427 | 1 | -0.601* | -0.126 |
| DOC | 0.839** | 0.608* | -0.622* | -0.406 | -0.520 | -0.350 | -0.601* | 1 | 0.699* |
| SOC | 0.706* | 0.371 | -0.399 | 0.203 | -0.535 | -0.322 | -0.126 | 0.699* | 1 |

The abbreviations are the same as those used in Table 1. Significance levels are marked as follow: * (*P* < 0.05) and ** (*P* < 0.01).
